# Endogenous oxytocin and intermale interactions after oxytocin administrations in Norway rats selected for behavior

**DOI:** 10.18699/vjgb-25-92

**Published:** 2025-10

**Authors:** S.G. Shikhevich, R.V. Kozhemyakina, R.G. Gulevich, Yu.E. Herbeck

**Affiliations:** Institute of Cytology and Genetics of the Siberian Branch of the Russian Academy of Sciences, Novosibirsk, Russia; Institute of Cytology and Genetics of the Siberian Branch of the Russian Academy of Sciences, Novosibirsk, Russia; Institute of Cytology and Genetics of the Siberian Branch of the Russian Academy of Sciences, Novosibirsk, Russia; Institute of Cytology and Genetics of the Siberian Branch of the Russian Academy of Sciences, Novosibirsk, Russia University of Haifa, Haifa, Israel

**Keywords:** oxytocin, selection, behavior, rat, aggressiveness, immunohistochemistry, hypothalamus, окситоцин, отбор, поведение, крыса, агрессивность, иммуногистохимия, гипоталамус

## Abstract

The neuropeptide oxytocin (OT) secreted by specialized neurons in the hypothalamus affects social behavior and aggression in various animal species in a dose-dependent manner. Our earlier studies showed that OT administration by nasal application to adult and adolescent Norway rat males selected for enhanced aggressive response to humans reduced aggression upon the opponent in the resident-intruder test. By contrast, OT administration to rats selected for tame behavior exerted no effect on behavior or even enhanced aggression. It was still unknown how selection for behavior affected the endogenous oxytocinergic system in rats. Here we study the populations of OT-containing cells in the paraventricular and supraoptic nuclei of the hypothalamus in intact tame and aggressive rats with regard to lateralization, as the hypothalamus is known to be functionally asymmetrical. We have also assessed blood OT changes after nasal OT application to rats selected for behavior. As it is known that the effect of OT on rat aggressiveness may depend on the basal level of the latter, we have analyzed the effect of OT administration on behavior in tame and aggressive rats interacting on neutral ground, where the aggressiveness of males manifests itself less than in the defense of territory in the resident-intruder test. The asymmetry in the numbers of OT-containing cells in the left and right halves of the paraventricular and supraoptic nuclei has been observed only in tame rats. The number of such cells in the right half of tame rats is greater than in aggressive. In contrast, the blood OT level in tame rats is significantly lower than in aggressive ones both in the intact animals and after OT administration. Oxytocin administration to aggressive rats shortens aggressive interactions and lateral threats and reduces the number of the latter as compared to animals of the same behavior pattern having received saline. This observation may point to an anti-aggressive effect of OT. In tame rats, though, OT administration increases the number of hind leg kicks and kicking duration. It appears that the differences in the endogenous OTergic system of hypothalamus found in this study are associated with both the behavior formed during selection and different responses to exogenous OT in tame and aggressive animals.

## Abstract (ru)

Известно, что нейропептид окситоцин (ОТ), секретируемый специализированными нейронами в гипоталамусе, оказывает дозозависимое влияние на социальное поведение и агрессию у различных видов животных. Ранее нами было показано, что у взрослых и неполовозрелых самцов серых крыс, селекционируемых на усиление агрессивного поведения по отношению к человеку, назальные аппликации окситоцина вызывали антиагрессивный эффект по отношению к оппоненту в тесте резидент–интрудер, в то время как у крыс, селекционируемых на ручное поведение, аппликации окситоцина или не влияли на поведение, или вызывали усиление агрессивности. Однако оставалось неизвестным, как влияет отбор по поведению на эндогенную окситоцинергическую систему у крыс. В данной работе исследовали количество содержащих окситоцин клеток в паравентрикулярном (ПВЯ) и супраоптическом (СОЯ) ядрах гипоталамуса у интактных ручных и агрессивных крыс, учитывая фактор латерализации, поскольку было известно о функциональной асимметрии гипоталамуса. Наряду с этим оценивали, как изменяется уровень окситоцина в крови после назальных аппликаций этого нейропептида у крыс, селекционируемых по поведению. Так как эффекты окситоцина на агрессивность крыс могут зависеть от степени ее проявления, в данной работе исследовали влияние аппликаций окситоцина на поведение у ручных и агрессивных крыс при взаимодействии на нейтральной территории, где агрессивность самцов проявляется слабее, чем при защите собственной территории в тесте резидент–интрудер. Показано, что только для ручных крыс характерна асимметрия по количеству содержащих окситоцин клеток, локализованных в правых и левых частях СОЯ и ПВЯ гипоталамуса. Причем количество таких клеток в правой половине СОЯ у ручных крыс оказалось больше, чем у агрессивных, в то время как уровень окситоцина в крови у ручных крыс как в контрольной группе, так и после аппликаций окситоцина, напротив, был достоверно ниже, чем у агрессивных. Аппликации окситоцина у агрессивных крыс вызывали уменьшение продолжительности агрессивных взаимодействий и боковых стоек угроз, а также количества последних по сравнению с животными того же поведения, получающими физраствор, что может свидетельствовать об антиагрессивном эффекте окситоцина, тогда как у ручных крыс аппликации окситоцина, наоборот, приводили к увеличению количества ударов задними лапами и их продолжительности. По-видимому, найденные нами различия в эндогенной окситоцинергической системе гипоталамуса могут быть связаны и с поведением, сформированным в процессе отбора, и с различной реакцией на введение окситоцина у ручных и агрессивных крыс.

## Introduction

Oxytocin (OT) had long been connected only with the reproductive function, maternal behavior, and nursing. Largescale studies of the entire range of OT’s physiological effects commenced in the mid-20th century. The results obtained in animals and, later, in humans, indicate that OT is essential for mitigating anxiety (Neumann, Slattery, 2016; Yoon, Kim, 2020; Takayanagi, Onaka, 2021) and aggression (Calcagnoli et al., 2013; de Jong, Neumann, 2018; Herbeck, Gulevich, 2019; Marsh et al., 2021) and for improving memory and learning (Aydogan et al., 2018).

The hypothalamic neuropeptide oxytocin is synthesized primarily in neurons of the paraventricular (PVN) and supraoptic (SON) hypothalamus nuclei. Then it is delivered to the posterior pituitary via axons and stored in vesicles until release to the systemic blood flow (Castel et al., 1984). Also, OT is transported via collateral branches of axons of the hypothalamo–neurohypophyseal tract to various parts of the forebrain, where OT receptors are expressed. There, it acts on various behavioral aspects (Jurek, Neumann, 2018; Grinevich, Neumann, 2021). Physiological stimuli, such as childbirth, lactation, stress, or emotions, induce a fast release of preaccumulated OT to blood and various parts of the brain (Eliava et al., 2016; Tang et al., 2020; Grinevich, Neumann, 2021).

The hypothalamus plays the key role in motivation formation and behavior triggering (Simonov 1987, 1993; Sudakov, 1993). The data on the functional hypothalamus asymmetry are scarce (Pavlova, 2001; Kiss, 2020). In particular, comparison of the efficiency of the stimulation of the right and left hypothalami in rabbits by the conjugated neuronal impulsation method in order to induce motivational and emotional responses showed that the left hypothalamus contributes more to defensive motivation, whereas the right hypothalamus, to emotional positive affects (Pavlova, 2001). The Norway rats selected for friendly and aggressive attitude to humans that were used in our studies differ significantly in the defense response to the experimenter’s glove. This difference may be associated with the location of OT-secreting neurons in the animals

I. Neumann et al. (2013) found that OT levels rose in blood and in dialysates of the dorsal hippocampus and amygdule of Wistar rat and С57Bl/6 mouse males 70 min after intranasal OT administration. They assume that the doses of exogenous OT in these brain regions were significant for behavior regulation.

F. Calcagnoli et al. (2014) examined Norway rats taken from the wild in Groningen (Netherlands), which differed from laboratory strains in greater and broadly variable maleto- male aggression. The scientists found that the OT mRNA level in PVN but not in SON inversely correlated with aggressiveness. The most aggressive males had lower OT mRNA levels in PVN than less aggressive ones. Oxytocin administration to the cerebral ventricular system of Norway male rats reduced their aggressiveness in a dose-dependent manner, which was most pronounced in males with higher aggression (Calcagnoli et al., 2013). Human studies also revealed differences in the efficiency of nasal OT administration on prosocial behavior (signs of positive interactions with other individuals) depending on the preexperimental social motivation in the subject. The improvement of prosocial behavior in response to intranasal OT administration was most pronounced in people with low social motivation, but exogenous OT could aggravate interpersonal anxiety in people with a low level of social safety (Bartz et al., 2015; Soriano et al., 2020).

Studies of Norway rats selected for aggressive and tolerant attitude to humans can help understand features of the functioning of the endogenous OTergic system and the role of OT in behavior regulation. The Norway rat population selected for tame behavior demonstrates a complete absence of defense responses and high tolerance of handling by humans. In contrast, the aggressive rats are characterized not only by high aggression toward humans but also by invariable and extreme intraspecies male-to-male aggression (Plyusnina, Solov’eva, 2010; Plyusnina et al., 2011).

It had been found that the activity of both the peripheral and central segments of the hypothalamic–pituitary–adrenal (HPA) axis in tame rats was higher than in aggressive or unselected animals (Plyusnina, Oskina, 1997; Herbeck et al., 2017). The aggression of male rats selected for aggressive behavior toward the opponent in the test on neutral ground was weaker than in the resident–intruder test (RIT), where animals under study defended their own space (Plyusnina, Solov’eva, 2010). Nasal OT administration to adult and adolescent aggressive males suppressed aggression in the RIT test. In tame rats, OT administration at the 2 μg/μL concentration to adult and adolescent males either exerted no effect on behavior or even enhanced signs of aggression (Gulevich et al., 2019; Kozhemyakina et al., 2020).

It is unknown how rat selection for behavior affects the parameters of the hypothalamus OTergic system and OT level in peripheral blood or whether the effect of OT applications on rat behavior will remain in the test on neutral ground, where male aggression is milder than in the RIT test.

In this view, the goal of the present study was to investigate features of the function of the endogenous OTergic system in tame and aggressive male rats and the effect of nasal OT administration on blood OT level and manifestation of aggression in the test on neutral ground

## Materials and methods

Experimental animals. Experiments were conducted at the conventional vivarium of the Institute of Cytology and Genetics (Novosibirsk) with adult male Norway rats (Rattus norvegicus). Two populations of the animals, hereafter referred to as tame and aggressive, had been selected for absence or enhancement of aggressive response to humans, respectively, for 84–93 generations. Animals were kept in metal cages, 50 × 33 × 20 cm, four males per cage, and exposed to artificial light schedule 12/12. Food and water were given ad libitum. All tests were done from 14:00 to 18:00 local time.

All international, national, and/or institutional principles of animal care and use were followed. All procedures in experiments with animals met the ethical standards mandated by Russian Federation legislation, principles of the Basel Declaration, and recommendations of the Bioethics Committee at the Institute of Cytology and Genetics of the Russian Academy of Sciences (protocol No. 8, dated March 19, 2012).

Immunohistochemical examination of SON and PVN in the hypothalamus of intact tame and aggressive rats. The experiment was conducted with rats of selection generation 89. Brains were fixed by perfusion through the aorta left ventricle with a peristaltic pump with phosphate-buffered saline (1×PBS) for 5–10 min and with 4 % paraformaldehyde for 5–10 min. The extracted brains were dehydrated by immersion in 30 % sucrose at +4 °C for 3–4 weeks and stored in Tissue-Tek® O.C.T. Compound at –70 °C until cryosection. Frontal sections (30 μm) were prepared in a Microm HM- 505 N cryostat (Microm, Germany) at –20 °C and mounted on Polysine® slides (Menzel-Gläser, Germany). The slides were stored at –20 °C until further examinations.

Slides were stained by the conventional method with the Rabbit specific HPR/DAB (ABC) Detection IHC kit ab64261 (Abcam, United Kingdom). Antibodies (Anti- Oxytocin- neurophysin 1 antibody ab2078 (Abcam)) were diluted 1/10,000. Slides were incubated with the antibodies for 3 days.

**Fig. 1.**
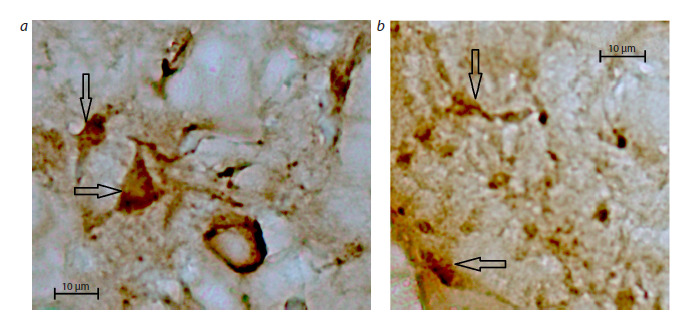
Immunohistochemical staining of oxytocin-containing cells in (a) paraventricular (PVN) and (b) supraoptical (SON) nuclei of the rat hypothalamus. Camera lens 40×.

Oxytocin-containing cells in SONs and PVNs in slides were counted under an AXIO optical microscope (Zeiss, Germany) (Fig. 1). Oxytocin-containing cells in PVNs were arranged into compact groups; we chose the region with the greatest density of stained cells. Its area was 4.7 μm2, and cells were counted there in all slides examined. We found no such specific region in SONs and determined the number of cells per 1 μm2 of the section

Nasal oxytocin applications and blood oxytocin assay thereafter. The experiment was conducted with rats of selection generation 93. Experimental groups included 10–12 animals. To minimize the stressing effect of the application of OT or saline, the animals had been habituated to handling for 7 days. On day 8, one group (OT group) received one application of 20 μL OT at the concentration of 2 μg/μL. Another group (saline group) received 20 μL of saline. Animals were decapitated 40 min after the procedure, and peripheral blood samples were taken to assay OT in the plasma. Intact rats were used as the control. Samples were taken into VACUETTE tubes (4 mL, 13 × 75 mm) with K3 EDTA and protease inhibitor aprotinin PREMIUM. Blood was centrifuged no later than 20 min after sampling. Plasma was immediately frozen at –20 °C and stored at –70 °C.

Oxytocin was assayed with DetectX® Oxytocin ELISA Kit (Arbor Assays, USA). To extract OT for assay, 200 mL of 0.05 M Tris HCl pH 8.0 and 5 μL of DTT (BioChemica, Pakistan) were added to 100 μL of plasma, and the mixture was incubated at 37 °C for 45 min. Iodoacetamide (0.5 M, 15 μL) was added, and the mixture was incubated at room temperature for 20 min. Then 640 μL of 80 % acetonitrile was added, and the mixture was centrifuged at 14,000g for 15 min. The supernatant was collected and dried with Concentrator plus (Eppendorf, Germany).

Study of male-to-male interactions on neutral ground. The experiment was conducted with rats of selection generation 84. Experimental groups included 9–10 animals. Two weeks before the experiment, animals were placed into individual cages. In one group (OT group), each rat received one application of 20 μL of 1 μg/μL OT onto epithelium around the nostrils. Another group received 20 μL saline (saline group). After 40 min, an experimental animal was placed into an unfamiliar cage (40 × 40 × 60 cm) divided into two equal compartments with a partition (Plyusnina, Solov’eva, 2010). Simultaneously, a Wistar male of close bodyweight was placed into the second compartment, and the partition was removed. The behavior was camcorded for further analysis. The test lasted for 10 min.

Agonistic behavior was assessed by the following parameters: latency of the first aggressive interaction, number and duration of attacks, chases, hind leg kicks, rearings, backfalls, aggressive grooming, and lateral threats (Plyusnina, Solov’eva, 2010; Soriano et al., 2020). The overall time of aggressive behavior included the duration of all these postures and movements associated with competition or conflict between animals. In addition, we assessed the overall time of social nonaggressive behavior, which included approaches and sniffings

Statistical evaluation. The results were evaluated with Statistica 10.0 (Stat Soft™, USA). Distribution normality was checked by the Kolmogorov–Smirnov test. Data on the numbers of OT-containing cells in hypothalamus nuclei and blood OT level, fitting into the normal distribution, were analyzed by parametric methods: Student’s test and ANOVA for independent measurements followed by post- hoc Fisher’s LSD test.

Data on the numbers of OT-containing cells in whole SONs and PVNs were analyzed by Student’s t-test, and the numbers in the left and right halves of SON and PVN were assessed by two-way ANOVA, where one of the factors was selection for behavior, and the other, lateralization, that is, whether the count referred to the right or left portion of SON or PVN.

Blood OT level data were assessed by two-way ANOVA, where one factor was selection for behavior and the other, application of OT or saline. The test of data on behavior features did not confirm distribution normality; therefore, we applied the nonparametric Mann–Whitney U-test for further processing. The data on behavior features are presented in the form of box-plots with maximum, minimum, and median values, where 50 % of the results for a given sample are within the box, 25 % from the minimum value to the box bottom, and 25 % from the maximum to the box top.

## Results

**Immunohistochemistry**

In our study, the number of OT-containing cells in the entire PVN did not differ significantly between tame and aggressive rats (774.76 ± 38.98 and 826.16 ± 35.80, respectively), whereas in the entire SON it tended to be greater in tame rats than in aggressive ones: 434.10 ± 28.76 vs. 331.68 ± 37.16 (p < 0.06).

Data on the numbers of OT-containing cells in separate halves of SON and PVN were processed with two-way ANOVA, where one factor was selection for behavior and the other, lateralization (count in the left or right half of SON or PVN).

**Fig. 2.**
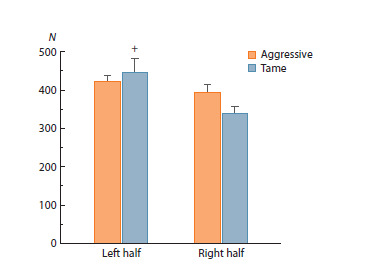
The numbers of oxytocin-containing cells in the left and right halves of rat PVNs. Two-way analysis of variance (ANOVA) followed by post-hoc Fisher’s test: + p < 0.05 as compared to the right PVN. N – number of cells.

The results indicate that selection for behavior did not affect the numbers of OT-containing cells in separate halves of PVN, whereas the effect of lateralization was significant (F1.65 = 8.08, p < 0.01). No interaction of these factors was observed. Post-hoc analysis showed that the number of OT- containing cells in the left half of PVN was greater than in the right in tame rats but not in aggressive ones (p < 0.05, Fig. 2).

**Fig. 3.**
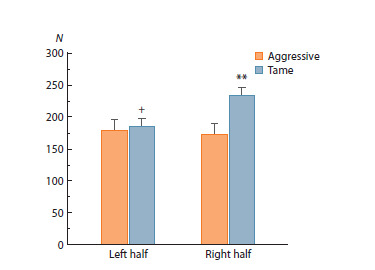
The numbers of oxytocin-containing cells in the left and right halves of rat SON. Two-way analysis of variance (ANOVA) followed by post-hoc Fisher’s test: ** p < 0.01 as compared to aggressive rats, + p < 0.05 as compared to the right half of SON. N – number of cells.

In contrast to PVN, the numbers of OT-containing cells in separate SON halves were significantly affected by the selection for behavior factor (F1.56 = 4.2, p < 0.05) but not by lateralization. The factor interaction was insignificant. According to the subsequent post-hoc analysis, the number of OT-containing cells in the right half of SON was significantly greater in tame rats than in aggressive ones ( p < 0.01, Fig. 3), which agrees with the aforementioned trend toward the greater number of these cells in the entire SON in tame rats as compared to aggressive ones. In addition, the number of OT-containing cells in the right half of SON in tame rats was significantly greater than in the left half ( p < 0.05).

**Blood oxytocin after nasal applications**

**Fig. 4.**
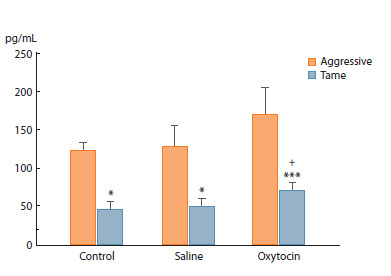
Oxytocin levels, pg/mL, in rat blood plasma 40 min after the application of OT (20 μL, 2 μg/μL) or saline (20 μL). Two-way analysis of variance (ANOVA) followed by post-hoc Fisher’s test: * p <0.05, *** p < 0.001 as compared to aggressive rats; + p < 0.05 as compared to the control.

Two-way ANOVA demonstrated a significant influence of rat selection for behavior on the OT level in blood plasma (F2.55 = 23.65, p < 0.001) but no influence of OT administration. No factor interaction was found either. The subsequent post-hoc analysis showed that blood plasma OT level in tame rats was significantly lower than in aggressive ones in all groups examined: p < 0.05 in the control and saline groups; p <0.001 in the OT group (Fig. 4). Saline applications did not affect blood OT in either tame or aggressive rats. The blood OT level in tame OT rats was higher than in the intact control ( p < 0.05).

**Effect of nasal oxytocin applications on rat behavior in the test for male-to-male interactions on neutral ground**

**Fig. 5.**
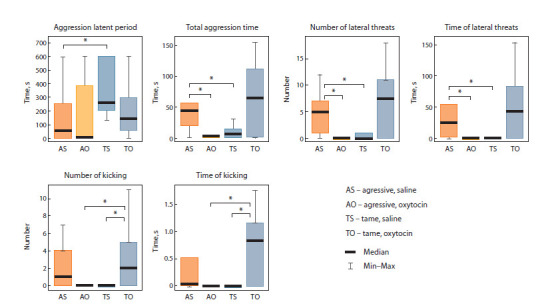
Effect of nasal oxytocin applications (20 μL, 1 μg/μL) on behavior patterns in the test for male-to-male interactions on neutral ground. * p <0.05, Mann–Whitney U-test.

Figure 5 presents data on behavior patterns in the test for male-to-male interactions on neutral ground, where significant differences were observed. In our experiments, the saline group of aggressive rats significantly exceeded the corresponding tame group in the overall time of aggressive interactions and the number and duration of lateral threats: p < 0.03, U = 17.5, Z = 2.2; p < 0.035, U = 18, Z = 2.16; p < 0.04, U = 19, Z = 2.08, respectively. The latency of aggression in aggressive animals after saline applications was significantly shorter than in tame ones: p < 0.02, U = 16, Z = –2.33.

Oxytocin applications to aggressive rats significantly reduced the overall time of aggressive interactions and the number and duration of lateral threats in comparison to rats of the same behavior group having received saline: p <0.04, U = 19, Z = 2.08; p < 0.035, U = 18, Z = 2.16; p < 0.025, U = 17, Z = 2.24, respectively. Tame rats having received OT showed a significant increase in the number and duration of hind leg kicks as compared to the corresponding saline group: p <0.02, U = 16, Z = –2.33 in both cases. Also, tame OT rats show a tendency toward longer aggressive interactions, and toward a greater number and duration of lateral threats than saline animals.

Comparison of behaviors in aggressive and tame rats after OT applications indicates that the number and duration of hind leg kicks in aggressive rats significantly decreased compared to tame ones: p < 0.02, U = 16, Z = –2.33 in both cases. Oxytocin administration leveled the differences between tame and aggressive rats in the overall time of aggressive interactions and the number and duration of lateral threats noted after saline administration

## Discussion

The immunohistochemical study of hypothalamus nuclei revealed an asymmetry in the numbers of OT-containing cells in SON and PVN in tame rats but not in aggressive ones. Data on the functional asymmetry of hypothalamus in rabbits obtained by conjugated neuronal impulsation in order to induce motivational and emotional responses indicate that the left hypothalamus contributed most to defensive motivation, and the right, to emotionally positive responses (Pavlova, 2001).

Our results indicate that the number of OT-containing cells in the right SON of rats selected for tame behavior is significantly greater than in aggressive ones (Fig. 2). As the rats selected for tame or aggressive behavior profoundly differ in the defense response to humans, we conjecture that the observed asymmetry in the numbers of OT-containing cells in the SON and PVN of tame rats and the greater number of such cells in the right half of SON in tame rats than in aggressive ones contribute to the difference in their attitude to humans.

The greater number of OT-containing cells in the right SON of tame rats as compared to aggressive ones is consistent with the observed tendency for the greater number of these cells in the entire SON ( p = 0.07). In other words, it is reasonable to suggest that the number of OT-containing cells inversely correlates with rat aggressiveness, which is generally consistent with lower aggressiveness in aggressive rats after OT administration by injection to a brain ventricle or by nasal applications (Calcagnoli et al., 2013, 2015; Gulevich et al., 2019).

Experiments with Groningen male rats reveal an inverse correlation between OT mRNA levels in PVN and animal aggressiveness (Calcagnoli et al., 2014). These data, as well as those obtained in our studies, indicate that the functional parameters of the endogenous OTergic system in the hypothalamus of more aggressive rats are lower than in less aggressive (in Groningen rats) or tame (in our selection model) animals. Also, although aggressive male rats differed in the number of OT-containing cells in the right SON in our study, the Groningen rat groups differing in aggressiveness differed in the levels of OT mRNA in PVN (Calcagnoli et al., 2014). These features in the location of differences in rats studied by us and in the Groningen rats differing in aggressiveness may be related to different approaches to the formation of aggressive behavior and its evaluation. The aggressiveness of Groningen rats was evaluated from the percentage of attack duration in ten RITs, and the aggressiveness in our study had been formed by long-term selection for attitude to humans.

It has been shown that blood OT in aggressive rats is significantly higher than in tame ones. As OT release to blood does not always correlate with local OT release from axon terminals in various regions of the brain (Knobloch et al., 2012; Grinevich et al., 2015), the assessment of brain OT system activity judging by blood OT level may be ambiguous, and OT variation in plasma does not necessarily correlate with animal behavior (Neumann, 2008). Forty minutes after nasal OT application, its blood level in tame rats was significantly higher than in intact animals but not higher than in the saline group, although this parameter did not differ significantly between the intact control and the saline group (Fig. 4). In Wistar rats, the blood OT level after OT application increased after 70, 100, and 130 min (Neumann et al., 2013). It seems that more time after nasal applications than in our study is required for OT elevation in blood and for the manifestation of its effects on behavior, which we observed as early as 40 min after applications both on neutral ground and in RIT (Gulevich et al., 2019; Kozhemyakina et al., 2020).

The behavior of aggressive rats in the test for male-tomale interactions on neutral ground points to an antiaggression effect of OT. The aggressive animals having received one OT application (20 μL, 1 μg/μL) differed from the saline aggressive group in showing significantly shorter aggressive interactions and lateral threats and in a smaller number of the latter. Similar effects of OT applications were noted in experiments with Groningen male rats in RIT (Calcagnoli et al., 2015) and in rats having been selected for aggressive behavior in interactions with an opponent male rat or with the experimenter’s glove (Gulevich et al., 2019; Kozhemyakina et al., 2020). In aggressive rats, aggression lowered after single applications of OT at the concentration of 2 μg/μL and after five daily applications of OT at a lower concentration, 1 μg/μL, that is, at OT concentrations varying within a certain range. As it had been shown that the aggression of aggressive rats on neutral ground was weaker than in animals defending their own territory in RIT (Plyusnina, Solov’eva, 2010), it is conceivable that OT mitigates aggression regardless of its manifestation in different tests.

Tests of tame rats for behavior on neutral ground revealed an opposite effect of OT applications (20 μL, 1 μg/μL): a significant increase in the number of hind leg kicks and kicking duration. Tame OT rats also showed trends toward longer aggressive interactions, longer lateral threats, and a greater number of the latter as compared to tame saline animals (Fig. 5). The change of these behavior parameters is indicative of aggressive behavior enhancement in tame rats. Previous data of RIT after 5-day OT applications at the same dose as in this study (20 μL, 1 μg/μL) showed not only more hind leg kicks in tame rats but also longer aggressive interactions, longer lateral threats, and more attacks (Gulevich et al., 2019). Single OT applications at the higher concentration of 2 μg/μL exerted no significant effect on tame rat behavior in RIT (Gulevich et al., 2019).

Further studies are needed for the understanding of this inverse effect of higher OT doses. The enhancement of certain aggressive behavioral acts in tame rats having received exogenous OT in the stressing situation of interaction with an opponent on neutral ground as compared to the control is consistent with data from other scientists. For instance, studies on domesticated animals (dogs, cattle, and pigs) and humans demonstrate that OT in stress can induce a paradoxical response to neutral or even affiliative (or positive) interaction by enhancing aggression and stress response (Rault et al., 2013; Hernádi et al., 2015; Yayou et al., 2015; Crespi, 2016; Shamay-Tsoory, Abu-Akel, 2016).

An autoradiographic study of Groningen rats displaying maximum aggressiveness showed that OT receptor expression in the central amygdule and bed nucleus of stria terminalis was higher than in less aggressive animals (Calcagnoli et al., 2014). As thought by the scientists, this fact partially compensates for the lower OT expression in PVN in aggressive rats and sensitizes them to exogenous OT. The expression of OT receptors in both brain regions correlated with the duration of offensive aggression in a 10-min RIT. Thus, it can be expected that the expression of OT receptors in rats selected for aggressive behavior in the central amygdule and bed nucleus of stria terminalis would also be higher than in tame animals. Further inquiry is needed to evaluate OT receptor expression in these brain regions in rats selected for behavior.

As mentioned above, the greatest prosocial effects of nasal OT administration to humans were observed in persons with poor social motivation or those avoiding social communication (Bartz et al., 2015; Soriano et al., 2020). In our study, the antiaggression OT effect was noted only in aggressive rats, the behavior of which is characterized not only by high aggressiveness toward humans but also by stable and extreme manifestation of intraspecific male-to-male aggression; thus, these rats can serve as models for studying mechanisms that underlie neuropsychiatric diseases – autism, social anxiety, and depression – and for elaborating approaches to their treatment.

Studies on rats and voles point to a functional relationship between the OTergic system and the HPA axis (Neumann et al., 2000; Engelmann et al., 2004; Smith, Wang, 2014). The elevated activity of the endogenous OTergic system in lactation or after social interactions is followed by lower ACTH and corticosterone secretion at rest and after stressing events, such as the presence of a conspecific opponent (Neumann et al., 2000; Engelmann et al., 2004; Smith, Wang, 2014). The inhibiting effect of OT on the HPA axis in stress may be mediated by downregulation of the Crh gene for the corticotropin-releasing hormone in the hypothalamus (Jurek et al., 2015). It is conceivable that the greater number of OT-containing cells in the right SON of tame rats than in aggressive ones is associated with the lower Crh expression in the hypothalamus, which had been found in our earlier studies of both intact animals and those having received OT applications (Herbeck et al., 2017; Gulevich et al., 2019).

## Conclusion

The mitigation or, in contrast, enhancement of aggressive behavior induced by exogenous OT in rats selected for behavior is determined by differences in the activity of the endogenous OTergic system and, probably, its functional relationship with the HPA axis, which is essential in behavior regulation and is altered considerably by selection for behavior

## Conflict of interest

The authors declare no conflict of interest.
